# Cross Regulation of Sirtuin 1, AMPK, and PPARγ in Conjugated Linoleic Acid Treated Adipocytes

**DOI:** 10.1371/journal.pone.0048874

**Published:** 2012-11-14

**Authors:** Shan Jiang, Wei Wang, Jess Miner, Michael Fromm

**Affiliations:** 1 Department of Molecular Genetics, University of Texas Southwestern Medical Center, Dallas, Texas, United States of America; 2 Department of Animal Science, University of Nebraska, Lincoln, Nebraska, United States of America; 3 Center for Biotechnology, University of Nebraska, Lincoln, Nebraska, United States of America; Boston University School of Medicine, United States of America

## Abstract

*Trans*-10, *cis*-12 conjugated linoleic acid (*t*10*c*12 CLA) reduces triglyceride (TG) levels in adipocytes through multiple pathways, with AMP-activated protein kinase (AMPK) generally facilitating, and peroxisome proliferator-activated receptor γ (PPARγ) generally opposing these reductions. Sirtuin 1 (SIRT1), a histone/protein deacetylase that affects energy homeostasis, often functions coordinately with AMPK, and is capable of binding to PPARγ, thereby inhibiting its activity. This study investigated the role of SIRT1 in the response of 3T3-L1 adipocytes to *t*10*c*12 CLA by testing the following hypotheses: 1) SIRT1 is functionally required for robust TG reduction; and 2) SIRT1, AMPK, and PPARγ cross regulate each other. These experiments were performed by using activators, inhibitors, or siRNA knockdowns that affected these pathways in *t*10*c*12 CLA-treated 3T3-L1 adipocytes. Inhibition of SIRT1 amounts or activity using siRNA, sirtinol, nicotinamide, or etomoxir attenuated the amount of TG loss, while SIRT1 activator SRT1720 increased the TG loss. SRT1720 increased AMPK activity while sirtuin-specific inhibitors decreased AMPK activity. Reciprocally, an AMPK inhibitor reduced SIRT1 activity. Treatment with *t*10*c*12 CLA increased PPARγ phosphorylation in an AMPK-dependent manner and increased the amount of PPARγ bound to SIRT1. Reciprocally, a PPARγ agonist attenuated AMPK and SIRT1 activity levels. These results indicated SIRT1 increased TG loss and that cross regulation between SIRT1, AMPK, and PPARγ occurred in 3T3-L1 adipocytes treated with *t*10*c*12 CLA.

## Introduction

Conjugated linoleic acid (CLA) reduces adiposity in human and mouse adipocytes [1]–[4], and the *trans*-10, *cis*-12 CLA (*t*10*c*12 CLA) isomer is capable of causing this response [5]. The rates of fatty acid oxidation and lipolysis increased in *t*10*c*12 CLA-treated 3T3-L1 adipocytes [6], while lipolysis increased and fatty acid biosynthesis decreased in *t*10*c*12 CLA-treated human adipocytes [7], [8]. Molecular responses to *t*10*c*12 CLA include the activation of AMP-activated protein kinase (AMPK) [9], integrated stress response (ISR; [10], [11]) or unfolded protein response (UPR; [12]), mitogen-activated protein kinase (MAPK) cascades [7], and attenuation of peroxisome proliferator-activated receptor γ (PPARγ) protein levels [3]. Treatment with *t*10*c*12 CLA requires nuclear factor kappa-B (NF-κB) for an inflammatory response [3], [4], [10], [13]–[15] that includes increased prostaglandin biosynthesis in human adipocytes [11], in mouse white adipose tissue [16], and 3T3L1 adipocytes [17]. Despite this progress in understanding of the pathways involved in the early perception of *t*10*c*12 CLA and the complex regulation of the subsequent responses, much remains unknown in this process.

AMPK is a central regulator of cellular energy levels that is activated by increases in the cellular AMP/ATP ratio, various cellular stresses [18], [19], or treatment of adipocytes [9] or mice [20] with *t*10*c*12 CLA or mixed isomers of CLA. AMPK activation requires phosphorylation at Thr172 [21], and two of the target proteins inhibited by phosphorylation by AMPK are acetyl-CoA carboxylase (ACC), and fatty acid synthase, two key enzymes in fatty acid biosynthesis. Through this and other mechanisms [18], [19], activated AMPK decreases lipogenesis, increases fatty acid oxidation, and increases lipolysis in adipocytes *in vitro* and *in vivo* [22]. Phenformin and metformin are structurally related chemicals that can be used to activate AMPK [18], [23]. Metformin increases TG loss in *t*10*c*12 CLA treated adipocytes, while compound C, a potent inhibitor of AMPK, attenuates TG loss in this system [9].

PPARγ is a ligand-activated nuclear receptor that regulates lipogenesis and is a key regulatory point for controlling inflammation in adipocytes [24]. PPARγ forms a complex with nuclear receptor corepressors 1 or 2 (NCoR1 or 2) in the absence of its bound ligand [25]. PPARγ transactivation activity is also reduced by phosphorylation at Ser112 by extracellular signal-regulated kinase (ERK), c-Jun N-terminal Kinase (JNK), or p38 MAPKs, and a phosphorylation-dependent sumoylation at K107 [26]. In addition, a non-genomic role for PPARγ is emerging, as a number of PPARγ-dependent processes are too rapid to involve transcriptional responses [27], [28]. The critical role of PPARγ in the response to *t*10*c*12 CLA is demonstrated by the attenuated responses that occur on addition of PPARγ agonists [11], [29], [30].

Protein deacetylation is emerging as an important mechanism for regulating energy balance [31], [32]. Of the two major classes of histone/protein deacetylases in mammals, the NAD-dependent class III sirtuins are structurally and enzymatically distinct from those of the zinc-dependent class I/II deacetylases [33]. Within the seven members of the sirtuin family, SIRT1 (sirtuin 1) is particularly involved in regulating cell energy metabolism, cell stress, and cell fate [34]. SIRT1 directly binds to NCoR1 and directly or indirectly to PPARγ to repress PPARγ transactivation activity, inhibit adipogenesis, and increase fat loss in adipocytes [35]. SIRT1 deacetylates liver kinase B1 (LKB1), facilitating the ability of LKB1 to phosphorylate AMPK, defining a SIRT1/LKB1/AMPK signaling pathway that provides one of the connections between SIRT1 and AMPK for regulating energy metabolism [36], [37]. SIRT1 also deacetylates NF-κB, thereby modulating NF-κB transactivation activity [38], [39]. Amongst the chemicals that affect the activity of sirtuins, the activator SRT1720 preferentially activates SIRT1 [40], while sirtinol and nicotinamide are used as general sirtuin inhibitors that do not inhibit class I/II deacetylases [41]. Nicotinamide is a direct product of the sirtuin deacetylation reaction that inhibits sirtuin enzymes as a non-competitive product inhibitor [42]. Etomoxir indirectly inhibits sirtuin activity as it inhibits fatty acid transport into mitochondria which prevents an increase in NAD^+^, an activator of sirtuin activity [43].

In this study, we analyzed the functional role of SIRT1 in the attenuation of TG levels in *t*10*c*12 CLA-treated 3T3-L1 adipocytes. Our objectives were to test whether SIRT1 was functionally required for robust triglyceride reduction, and whether SIRT1, AMPK, and PPARγ cross regulated each other in the response. These experiments were performed with activators, inhibitors, or siRNA that affected these pathways and analyzing their effects on TG levels, fatty acid metabolism, and post-translational modifications or activity levels of SIRT1, AMPK, and PPARγ.

## Experimental Procedures

### Reagents

Compound C was purchased from Calbiochem (San Diego, CA). Bovine serum albumin (BSA, >99% fat free), dexamethasone, etomoxir, GW9662, insulin, isobutyl-1-methylxanthine, metformin, nicotinamide, phenformin, and sirtinol were purchased from Sigma (St. Louis, MO). Ciglitazone, pioglitazone, rosiglitazone, and troglitazone were from Fisher (Pittsburgh, PA). SRT1720 was from Cayman Chemical (Ann Arbor, MI). T10c12 CLA (90%, #UC-61-A) was from Nu-Chek Prep, Inc (Elysian, MN). Antibodies to acetyl-NF-κB p65 (acetyl K310), p-PPARγ (phospho S112) and negative control siRNA were from Abcam (Austin, TX). Protein A agarose beads, antibodies to β-actin, NCoR1, NF-κB p65, PPARγ, SIRT1, anti-goat or anti-rabbit secondary antibodies coupled to horseradish peroxidase, and Sirt1 siRNA were purchased from Santa Cruz Biotechnology (Santa Cruz, CA). Antibodies to p-AMPK, AMPK, p-ACC, and ACC were from Cell Signaling (Beverly, MA). T7 RNA polymerase (P2077) and rNTPs (E6000) were purchased from Promega. SiRNA to lamin A/C, non-target siRNA, and DharmaFECT Duo transfection reagent (T-2010-02) were from Dharmacon (Thermo Fisher Scientific, Boulder, CO).

### 3T3-L1 cell culture, differentiation, and chemicals

Low passage 3T3-L1 fibroblasts [44] were obtained (H. Green, Harvard Medical School) and cultured in Dulbecco's modified Eagle's medium (DMEM; Invitrogen, Carlsbad, CA) containing 10% bovine calf serum (Fisher, Pittsburgh, PA) and differentiated as described [9]. When present, chemicals were dissolved in DMSO, with the exception that 2 mmol/L metformin and 0.1 mmol/L phenformin were dissolved in water, and were added directly to the media at ≤0.2% of the final volume in the media. An initial chemical concentration was determined from literature values and a range of concentrations around this value were then tested for their effects *in vivo*. From this data, the lowest effective concentration was chosen and used at the following concentrations: 5 µmol/L ciglitazone, 10 µmol/L compound C, 10 µmol/L etomoxir, 10 µmol/L GW9662, 10 µmol/L nicotinamide, 5 µmol/L pioglitazone, 5 µmol/L rosiglitazone, 10 µmol/L sirtinol, 8 µmol/L SRT1720, or 5 µmol/L troglitazone and were added 1 h before adding fatty acids. Fatty acids, either linoleic acid or trans-10, cis-12 CLA, were dissolved in 0.1 M KOH, diluted into fatty acid free (>99%) bovine serum albumin (BSA) in phosphate buffered saline at a 1∶1 ratio (2 mmol/L BSA: 2 mmol/L fatty acid), pH adjusted to 7.4, and added to the cultures containing 4 to 6 d post-differentiated 3T3-L1 adipocytes [9]. We used 50 µM *t*10*c*12 CLA if assaying chemicals that increased TG loss, but otherwise used 100 µM *t*10*c*12 CLA.

### Fatty acid biosynthesis, oxidation, and lipolysis assays

Fatty acid biosynthesis was measured in differentiated adipocytes after 24 h of treatment by removing the treatment media and incubating the adipocytes in Hanks' Balanced Salt Solution (HBSS; Invitrogen, Carlsbad, CA) containing 37 KBq [^14^C]-acetate [specific activity 2.1 GBg/mmol, (PerkinElmer Radioisotopes, Waltham, MA)] for 30 min (incorporation was linear for 60 min). Cells were washed in PBS three times, pelleted, and then resuspended in 100 µl PBS and 0.1% SDS. Lipids were extracted in 1 ml of 2∶1 chloroform∶methanol [45] and measured by scintillation counting. Cells briefly exposed to 37 KBq [^14^C]-acetate, followed by immediate washing and extracted as above, were used to determine background levels, which were subtracted from sample values. Fatty acid oxidation was measured in differentiated adipocytes in 3.5 cm culture plates 12 h after starting treatments by adding 37 KBq [^14^C]-oleic acid [specific activity 2.2 GBg/mmol, (PerkinElmer Radioisotopes, Waltham, MA)] to the treatment media for 2 h and collecting [^14^C]-CO_2_ for 1 h in collection jars as reported [46]. For lipolysis assays, the TG pool of differentiated adipocytes was labeled by adding 37 KBq [^14^C]-acetate [specific activity 2.1 GBg/mmol, (PerkinElmer Radioisotopes, Waltham, MA)] to the media for 4 h, after which time the plates were washed four times with PBS, and specific experimental media treatments were initiated. Media (0.1 ml) was collected after 24 h, lipids extracted in 1 ml of 2∶1 chloroform∶methanol [45] and measured by scintillation counting. The use of labeled [^14^C]-acetate and the 2∶1 chloroform∶methanol extraction step considerably reduced non-specific background to 50 DPM, as determined by using the above protocol on cells that had been briefly exposed to 37 KBq [^14^C]-acetate in media.

### siRNA transfections

For siRNA transfections, 3T3-L1 adipocytes, 4 to 5 d post differentiation, were transfected by siQUEST transfection reagent (Mirus, Madison, WI) or DharmaFECT Duo transfection reagent (Dharmacon, Thermo Fisher Scientific, Boulder, CO) as described [47]. For siQUEST transfections, concentrations of 2 µl of siQUEST reagent per ml of media and 40 nmol/L of siRNA were added 24 h before adding fatty acids. For DharmaFECT Duo transfections, concentrations of 1.4 µl per cm^2^ of DharmaFECT Duo and 80 nmol/L of siRNA were added 24 h before adding fatty acids.

### T7 transcription of oligonucleotide templates

In addition to commercially available siRNAs, we utilized siRNAs derived from transcription of oligonucleotide templates with T7 RNA Polymerase as described [48]. The siRNA sequences used were: siRNA control sequence: 5′-AAC AGU CGC GUU UGC GAC UGG UCU CUU GAA CCA GUC GCA AAC GCG ACU GCC UAU AGU GAG UCG UAU UA-3′. LaminA/C siRNA sequence: 5′-AAG GAG GAG CUU GAC UUC CAG UCU CUU GAA CUG GAA GUC AAG CUC CUC CCC UAU AGU GAG UCG UAU UA-3′. Sirt1 siRNA: 5′-AAG GAG ACU GCG AUG UUA UAA UCU CUU GAA UUA UAA CAU CGC AGU CUC CCC UAU AGU GAG UCG UAU UA-3′

### Immunoblot analysis

Nuclear and cytosolic extracts were isolated using a nuclear extract kit (Active Motif, Carlsbad, CA). Equal amounts of proteins from whole cell, nuclear, or cytosolic extracts were separated by SDS-PAGE, transferred to Immun-blot PVDF membrane (Bio-Rad Laboratories, Hercules, CA), probed with the indicated primary antibodies, and detected with secondary antibodies. Enhanced chemiluminescence (Pierce, Rockford, IL) was used for detection. Band intensities were determined from digital images from exposures in the linear range using software (Quantity One, Biorad, Hercules, CA). All western blot analyses were repeated at least three times.

### Immunoprecipitation

Immunoprecipitations were performed according to the procedure described [35]. In brief, the collected 3T3-L1 adipocytes were sonicated, lysates were centrifuged, and aliquots of the supernatants were immunoprecipitated overnight with specific antibody or control nonspecific IgG serum. Protein A agarose beads were used to bind the specific or non-specific antibody complexes, the protein A beads containing bound proteins were washed five times, and the bound proteins were eluted in SDS sample buffer for immunoblot analysis.

### Quantification of TG content

Cell isolation and TG measurements were performed according to the manufacturer's instructions using TG reagent (T2449; Sigma, St. Louis, MO) and free glycerol reagent (F6428; Sigma, St. Louis, MO). TG data are expressed as nmol of TG per mg of protein.

### Measurement of MCP-1 and COX2 mRNA

Total RNA was extracted by TRIzol (Invitrogen) following the manufacturer's protocol. Total RNA (2 µg) was used for cDNA synthesis. Real-time PCR was performed by a Bio-Rad iCycler using iQ SYBR Green Supermix reagent (Bio-Rad, Hercules, CA) using PCR primers for MCP-1 or glyceraldehyde 3-phosphate dehydrogenase (GAPDH) [9], and COX2 [9]. MCP-1 and COX2 mRNA levels were normalized to GAPDH, which showed no significant variation in microarray analyses between linoleic acid and *t*10*c*12 CLA treatments. Experiments were repeated three times, and each sample was analyzed using one RNA control and two replicates of each cDNA pool, and the relative amounts of MCP-1, COX2 and GAPDH were calculated using the comparative C_T_ method [49], according to the manufacturer's software (Bio-Rad, Hercules, CA). Cycle numbers were used to calculate gene expression levels in the log linear amplification range.

### Statistical Analysis

One or two-way ANOVA was used to analyze the data. Post-hoc pairwise comparisons were calculated using Tukey's test and were considered significant for *p*≤0.05. All analyses were performed using SAS software (SAS, Cary, NC).

## Results

### SIRT1 is required for reduced TG levels in *t*10*c*12 CLA-treated adipocytes

**Figure 1:**
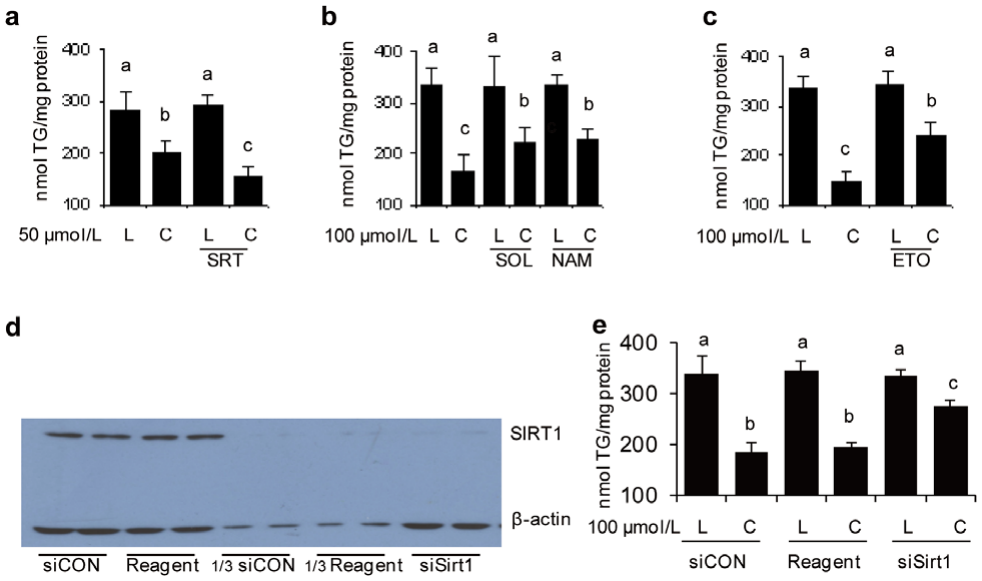
SIRT activity affects TG levels in *t*10*c*12 CLA treated 3T3-L1 adipocytes. **a–c** Triglyceride (TG) levels were measured in differentiating adipocytes after incubation with LA (L) or *t*10*c*12 CLA (C), with or without the SIRT1 activator SRT1720 (SRT), or the sirtuin inhibitors sirtinol (SOL), nicotinamide (NAM), or etomoxir (ETO) for 24 h. **d** Immunoblot analysis of whole cell extracts for the amount of SIRT1 and β-actin proteins after exposure to 80 nmol/L of control siRNA (siCON), or siRNA against SIRT1 (siSirt1), or transfection reagent only. Duplicate loadings of each sample were analyzed and the lanes marked by 1/3 have one third of the indicated samples loaded. **e** Triglyceride (TG) amounts after treatments as in **d**. Each bar in panels **a–c** and **e** represents the mean + SEM (n = 3), and is representative of three independent experiments. Means not sharing a common letter differ, *P* 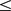
0.05. Panel d is a representative blot of three independent experiments.

We first determined whether increased SIRT1 activity would affect TG levels in *t*10*c*12 CLA-treated differentiating adipocytes. To specifically activate SIRT1, we used SRT1720, as this chemical is known to preferentially activate SIRT1 at 8 µM [40]. SRT1720, in combination with 50 µM *t*10*c*12 CLA, significantly lowered TG to levels below those achieved by 50 µM *t*10*c*12 CLA alone (Fig. 1A). In contrast, addition of 100 µM *t*10*c*12 CLA in combination with a sirtuin inhibitor, either sirtinol or nicotinamide, significantly increased TG levels, relative to those from adipocytes treated with 100 µM *t*10*c*12 CLA alone (Fig. 1. B). Etomoxir, which inhibits sirtuin activity indirectly by reducing NAD^+^ levels via inhibition of mitochondrial fatty acid transport and oxidation [43], was also tested. Treatment with etomoxir and *t*10*c*12 CLA significantly increased TG levels relative to TG levels with *t*10*c*12 CLA alone (Fig. 1C). These data supported a hypothesis that one or more sirtuins participate in the TG loss response caused by *t*10*c*12 CLA treatment, and that SIRT1 involvement was likely due to the response to SRT1720, a specific SIRT1 activator.

SiRNA was used to reduce SIRT1 expression to confirm the functional involvement of SIRT1 in the response to *t*10*c*12 CLA. First, to verify that SIRT1 protein levels were likely to respond to siRNAi-mediated knockdown of its mRNA levels in *t*10*c*12 CLA-treated adipocytes, the half-life of SIRT1 in cycloheximide treated adipocytes was determined to be less than 6 h (Figure S1) and that treatment with *t*10*c*12 CLA did not change SIRT1 protein levels (Figure S2). We additionally verified that our siRNA transfection method could deliver siRNA efficiently enough to knockdown protein levels of Lamin A/C (Figure S3), a target known to be susceptible to siRNA knockdown in adipocytes [47].

A siRNA targeted against SIRT1 mRNA significantly reduced SIRT1 protein levels but did not affect β-actin levels, while a control non-target siRNA did not change SIRT1 or β-actin amounts (Fig. 1D). In the presence of *t*10*c*12 CLA, siRNA knockdown of SIRT1 in adipocytes produced higher levels of TG levels than those from adipocytes treated with a non-target siRNA or reagent only controls (Fig. 1E). In the presence of LA, the siRNA SIRT1 treated adipocytes had TG levels that were not significantly different than those from adipocytes treated with the control non-target siRNA or reagent (Fig. 1E). These results indicate that siRNA knockdown of SIRT1 attenuated the TG loss caused by *t*10*c*12 CLA. Taken together with the above inhibitor studies, these results demonstrate that inhibition of SIRT1 activity or protein levels significantly interfere with the TG loss caused by *t*10*c*12 CLA. Further, the magnitude of the change in TG levels in the inhibitor studies (Figures 1b and 1c) and when SIRT1 was knocked down by siRNA (Figure 1e) indicates SIRT1 accounts for most or all of the SIRT activity involved in lipid loss in *t*10*c*12 CLA treated adipocytes.

### SIRT1 affects the rates of fatty acid metabolism and the inflammatory response

**Figure 2:**
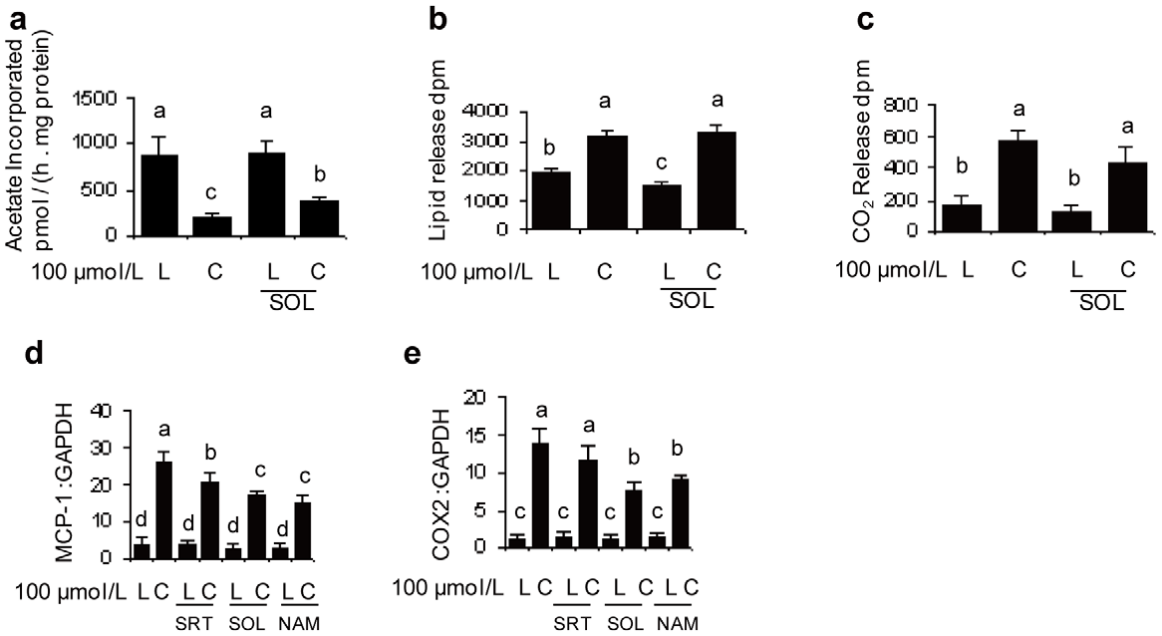
SIRT1 affects fatty acid metabolism and inflammatory mRNA levels. **a–c** The effects of 100 µmol/L of *t*10*c*12 CLA, with or without the sirtuin inhibitor sirtinol (SOL), on the rates of fatty acid metabolism were measured by radioactive tracers (disintegrations per minute: dpm) as follows: **a** fatty acid biosynthesis by incorporation of [1-^14^C]-acetate; **b** lipolysis by the release of lipids previously labeled with [1-^14^C]-acetate; **c** fatty acid oxidation by the release of [^14^C]-CO_2_ from lipids previously labeled with [1-^14^C]-oleate. **d–e** The effects of the SIRT1 activator SRT1720 (SRT), or the sirtuin inhibitors sirtinol (SOL) or nicotinamide (NAM) on RNA levels in LA or *t*10*c*12 CLA treated adipocytes were analyzed for MCP-1 and COX2 relative to GAPDH by reverse transcription and quantitative PCR. The relative amounts of MCP-1 and COX2 mRNA are shown as bar graphs. Each bar in panels **a–e** represents the mean + SEM (n = 3 for **a–b** or n = 2 for **c–e**), and is representative of three independent experiments. Means not sharing a common letter differ, *P* 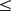
0.05.

The involvement of SIRT1 in *t*10*c*12 CLA-mediated changes in the rates of fatty acid biosynthesis, oxidation, and lipolysis as well as in the induction of two key inflammatory mRNAs was then measured to gain insight into how SIRT1 affected these specific pathways. Adipocytes treated with *t*10*c*12 CLA alone had a 77% reduction in their rate of lipogenesis (Fig. 2A). The combination of *t*10*c*12 CLA and sirtinol significantly changed this to a 47% reduction in the rate of lipogenesis (Fig. 2A). This result indicated SIRT1 activity was involved in inhibiting the rate of fatty acid biosynthesis. Adipocytes treated with *t*10*c*12 CLA exported significantly more radioactively-labeled lipids than LA treated cells, but the amount of *t*10*c*12 CLA-mediated lipolysis was not significantly affected by the sirtuin inhibitor sirtinol (Fig. 2B). The rate of fatty acid oxidation was significantly increased in *t*10*c*12 CLA-treated adipocytes, and although fatty acid oxidation was less when sirtinol was added, this difference was not significant (Fig. 2C). In conclusion, it appears the major change in lipid metabolism affected by SIRT1 is the rate of fatty acid biosynthesis.

The inflammatory MCP1 and COX2 mRNAs were highly induced by *t*10*c*12 CLA treatment (Fig. 2D–E). The SIRT1 activator SRT1720, in combination with *t*10*c*12 CLA, modestly attenuated the induction of MCP1 to 79% of *t*10*c*12 CLA levels, and although COX2 mRNA levels were slightly lower, this latter difference was not significant (Fig. 2D–E). In contrast, the SIRT1 inhibitors, sirtinol or nicotinamide, when used in combination with *t*10*c*12 CLA, attenuated the induction of the mRNA of MCP1 relative to *t*10*c*12 CLA control levels (Fig. 2D). Similarly, sirtinol or nicotinamide, when used in combination with *t*10*c*12 CLA, attenuated the induction of the mRNA of COX2 relative to 10*c*12 CLA control levels (Fig. 2E). These results indicated SIRT1 partially increased the induction of the inflammatory MCP1 and COX2 mRNAs, possibly through its inhibition of PPARγ (see below), which plays an anti-inflammatory role in adipocytes [24].

### SIRT1 increases AMPK activity

**Figure 3:**
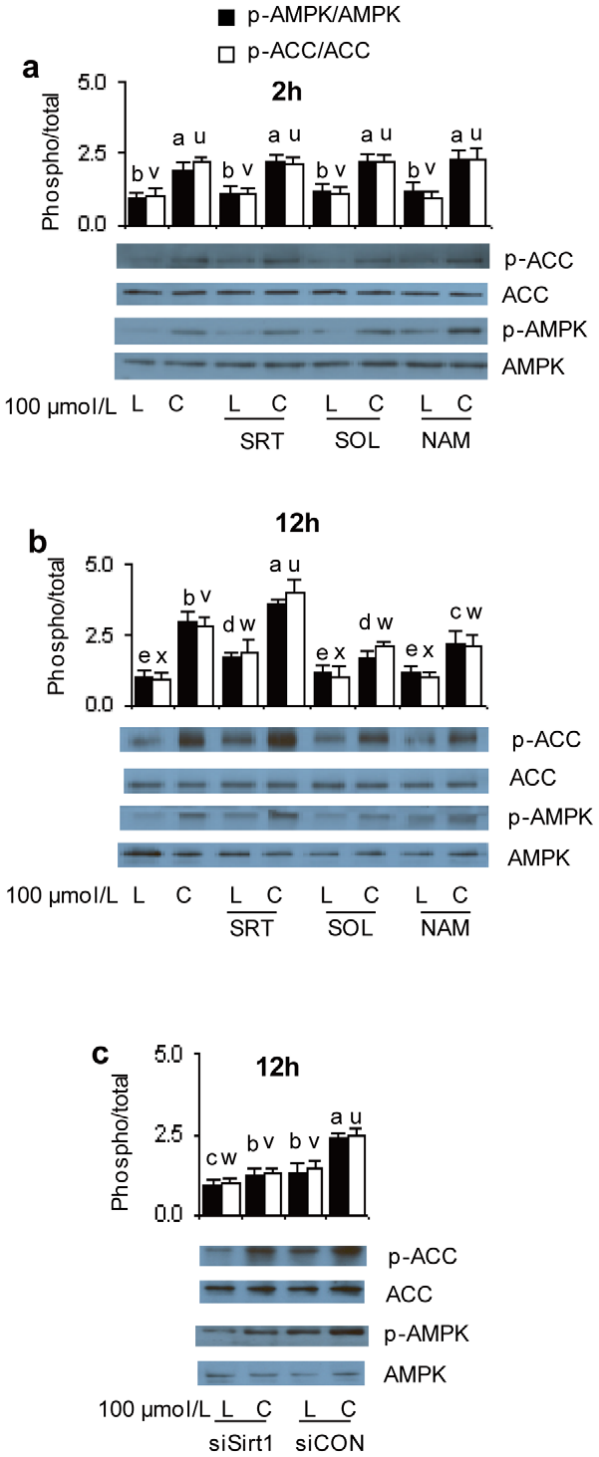
SIRT1 affects ACC and AMPK phosphorylation levels after *t*10*c*12 CLA treatment. Differentiated 3T3-L1 adipocytes were incubated with 100 µmol/L LA (L) or *t*10*c*12 CLA (C): **a** with or without SIRT1 activator SRT1720 (SRT), or sirtuin inhibitors sirtinol (SOL) or nicotinamide (NAM) for 2 or **b** 12 h; or **c** with 40 nmol/L of SIRT1 siRNA (siSIRT1) or control siRNA (siCON). Representative western blots indicate the proteins detected in cytosolic extracts with antibodies to p-ACC (p-Ser79), or total ACC, p-AMPK (p-Thr172), or total AMPK. Each bar in panels **a–c** represents the mean + SEM (n = 3) of the ratio of the phosphorylated to total form of each protein (phospho/total) for three independent experiments. Means for p-AMPK/AMPK (a–e) or p-ACC/ACC (u–x) not sharing a common letter differ, *P* 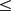
0.05.

We next determined whether SIRT1 affected AMPK regulation during the response to *t*10*c*12 CLA. AMPK activity was measured by the amount of phosphorylation at AMPK Thr172 (p-AMPK) and by the amounts of phosphorylated ACC (p-ACC), one of AMPK's key substrates *in vivo* [50]. We previously demonstrated the *t*10*c*12 CLA-stimulated increase in ACC phosphorylation was less in compound C treated adipocytes, supporting the premise that this phosphorylation was mediated by AMPK [9]. Although p-AMPK levels were increased by 2 h of treatment with *t*10*c*12 CLA, the addition of SIRT1 activator SRT1720 or SIRT inhibitors, had no significant effect on the amount of p-AMPK or p-ACC produced after 2 h of treatment (Fig. 3A). However, when used in combination with *t*10*c*12 CLA for 12 h, SIRT1 activator SRT1720 significantly increased p-AMPK or p-ACC levels relative to the amounts present when treated by *t*10*c*12 CLA alone (Fig. 3B). Conversely, when used in combination with *t*10*c*12 CLA for 12 h, SIRT1 inhibitors sirtinol or nicotinamide significantly attenuated p-AMPK levels relative to the amounts of these proteins in adipocytes treated with *t*10*c*12 CLA (Fig. 3B). Similarly, when used in combination with *t*10*c*12 CLA, siRNA targeting of SIRT1 significantly reduced p-AMPK and p-ACC levels relative to the control treatment (Fig. 3C). Collectively, these results indicated SIRT1 increased AMPK activity levels after 12 h, but not 2 h, of exposure to *t*10*c*12 CLA.

### A PPARγ agonist or antagonist affects the TG loss response to *t*10*c*12 CLA

**Figure 4:**
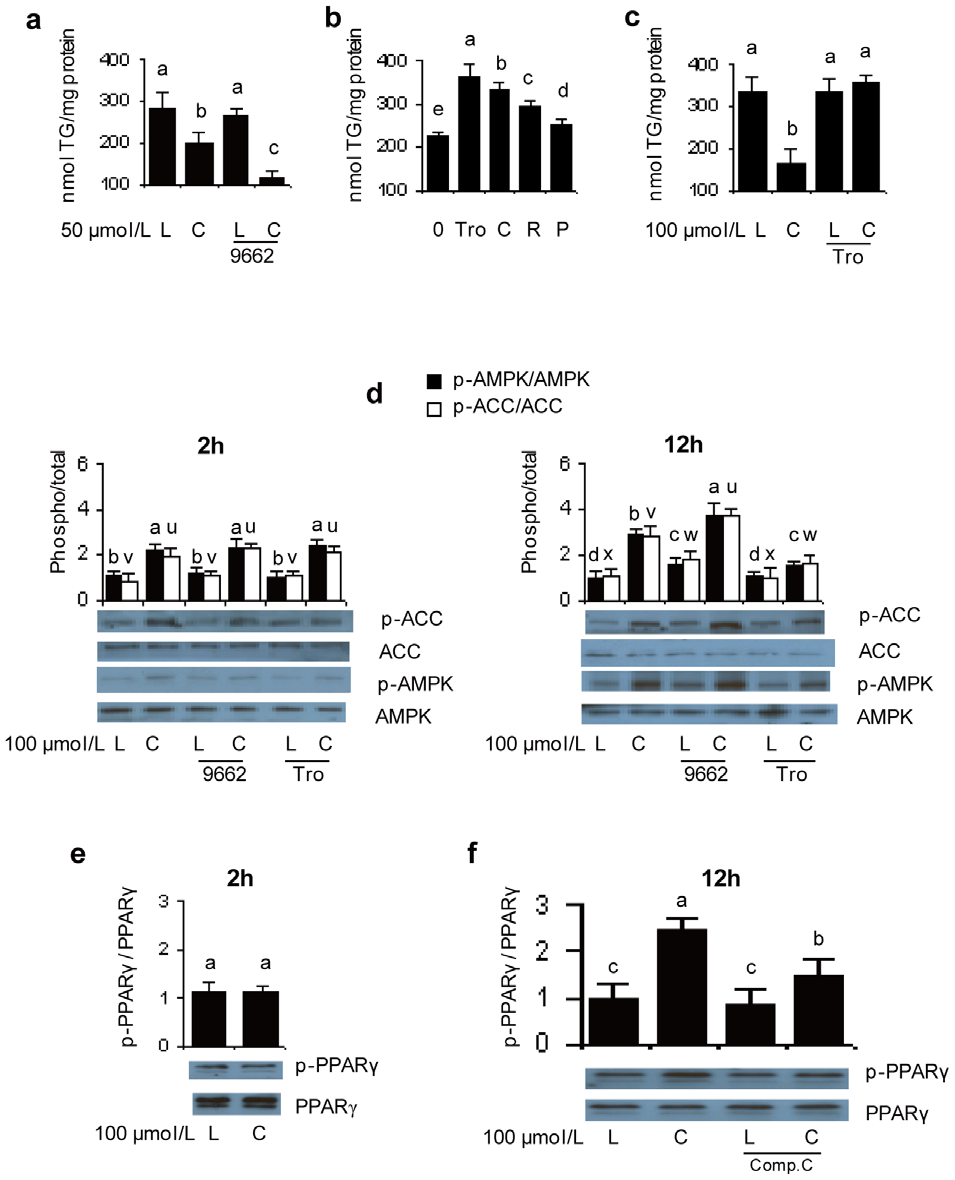
PPARγ antagonists and agonists affect TG levels and modulate p-AMPK activity levels in *t*10*c*12 CLA treated 3T3-L1 adipocytes. **a** Differentiated adipocytes were incubated with 50 µmol/L LA (L) or *t*10*c*12 CLA (C) with or without PPARγ antagonist GW9662 (9662) for 24 h and TG levels were measured. **b** Differentiating adipocytes were treated with either control media (0), troglitazone (Tro), ciglitazone (C), rosiglitazone (R), or pioglitazone (P) to determine which PPARγ agonist was most effective for increasing TG levels. **c** Differentiated 3T3-L1 adipocytes were incubated with 100 µmol/L LA or *t*10*c*12 CLA, with or without PPARγ agonist troglitazone (Tro), and TG levels were measured after 24 h. **d** Adipocytes were treated as in **a** or **c** for 2 or 12 h using 100 µmol/L LA or *t*10*c*12 CLA, and representative western blots of cytosolic extracts indicate the proteins detected with antibodies to p-ACC (p-Ser79), total ACC, p-AMPK (p-Thr172), or total AMPK. The ratio of the phosphorylated to total form of each protein (phospho/total) is shown in the bar graphs. **e–f** The amount of phosphorylated or total PPARγ in nuclear extracts was measured after 2 or 12 h of treatment with 100 µmol/L LA or *t*10*c*12 CLA, and with or without compound C (Comp. C) at 12 h. The ratio of the amount of phosphorylated to total PPARγ is shown in the bar graphs. Each bar in panels **a–f** represents the mean + SEM (n = 3), and is representative of three independent experiments (**a–c**) or is the mean of three independent experiments (**d–f**). Means within each data type (a–e or u–x) not sharing a common letter differ, *P* 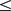
0.05.

The involvement of PPARγ in the TG loss response to *t*10*c*12 CLA was then tested through addition of a PPARγ antagonist or agonist. The PPARγ antagonist GW9662 significantly reduced TG levels when used in combination with 50 µM *t*10*c*12 CLA relative to TG levels in the *t*10*c*12 CLA treatment (Fig. 4A). Prior to examining the effects of a PPARγ agonist on the response to *t*10*c*12 CLA, we first determined that troglitazone was the most potent PPARγ agonist amongst a set of four thiazolidinedione agonists, as measured by their ability to increase the amount of TG produced in differentiating adipocytes (Fig. 4B). Troglitazone significantly attenuated *t*10*c*12 CLA's ability to reduce TG levels, as the combined treatment was not significantly different from the LA control (Fig. 4C). These results indicated that, in adipocytes treated with *t*10*c*12 CLA, a PPARγ antagonist facilitated TG loss while a PPARγ agonist interfered with TG loss.

### Cross regulation of PPARγ and AMPK in adipocytes treated with *t*10*c*12 CLA

The cross regulation between PPARγ and AMPK was next determined. When used in combination with *t*10*c*12 CLA for 12 h, PPARγ antagonist GW9662 significantly increased AMPK and ACC phosphorylation levels (Fig. 4D). Conversely, when used in combination with *t*10*c*12 CLA for 12 h, troglitazone significantly reduced AMPK and ACC phosphorylation levels (Fig. 4D). However, when used in combination with *t*10*c*12 CLA for only 2 h there was no significant effect of these chemicals on AMPK or ACC phosphorylation levels (Fig. 4D). Therefore, although AMPK activation occurred at 2 h, cross regulation by PPARγ was not apparent at 2 h. Phosphorylation of PPARγ increased by 140% at 12 h, but not by 2 h, after *t*10*c*12 CLA treatment (Fig. 4E–F). When compound C was used in combination with *t*10*c*12 CLA, the amount of phosphorylated PPARγ was reduced relative to the levels when treated by *t*10*c*12 CLA. This suggests AMPK activity was directly or indirectly involved in the phosphorylation of PPARγ (Fig. 4F). These results indicated AMPK and PPARγ cross regulated each other in the response to *t*10*c*12 CLA.

### Cross regulation of PPARγ and AMPK also occurs in the absence of *t*10*c*12 CLA

**Figure 5:**
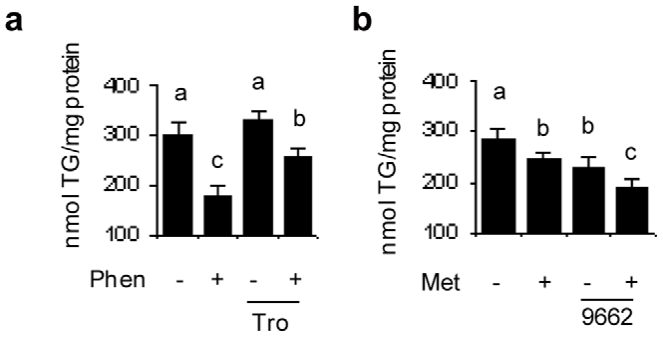
PPARγ agonists or antagonists affect the TG loss caused by AMPK activators without *t*10*c*12 CLA being present. **a** Differentiated 3T3-L1 adipocytes were incubated with or without 0.1 mmol/L phenformin (Phen), with or without PPARγ agonist troglitazone (Tro), and TG levels were measured after 24 h. **b** TG levels were measured in differentiated 3T3-L1 adipocytes in media lacking or containing 2 mmol/L metformin (Met), with or without PPARγ antagonist GW9662 (9662) for 24 h. Each bar represents the mean + SEM (n = 3), and is representative of three independent experiments. Means not sharing a common letter differ, *P* 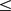
0.05.

We next determined whether this cross regulation between AMPK and PPARγ was a general aspect of these proteins by testing whether this occurred in the absence of *t*10*c*12 CLA. This was done by using two other chemicals to activate AMPK, alone or in combination with a PPARγ agonist or antagonist. Phenformin, a strong AMPK activator, significantly reduced TG levels to 60% of those present in the untreated control adipocytes (Fig. 5A). When phenformin was used in combination with troglitazone, significantly more TG was present than in adipocytes treated with phenformin alone (Fig. 5A). Metformin, a weaker AMPK activator, only slightly reduced TG levels (Fig. 5B). The TG level was further reduced when metformin was used in combination with the PPARγ antagonist GW9662 (Fig. 5B). These results indicated that the antagonistic cross regulation between AMPK and PPARγ that was observed in the response to *t*10*c*12 CLA also occurred in phenformin or metformin treated adipocytes in the absence of *t*10*c*12 CLA.

### NF-κB is deacetylated by one or more sirtuins in *t*10*c*12 CLA treated adipocytes

**Figure 6:**
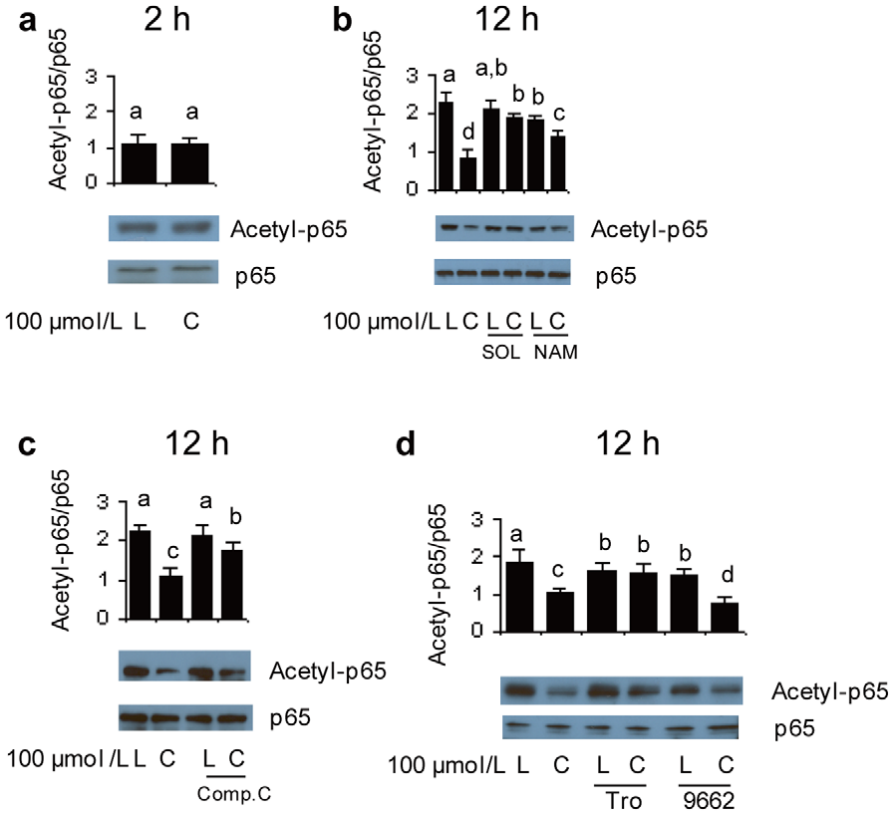
SIRT1, AMPK and PPARγ affect the *t*10*c*12 CLA-dependent decrease in the amount of acetylated NF-κB. The amount of acetylation on the p65 subunit of NF-κB or the total amount of p65 in nuclear extracts was detected by immunoblot analysis with antibodies specific for acetylated p65 or total p65. The ratio of the acetylated to total p65 is shown in the bar graphs (Acetyl-p65/p65). **a–c** Differentiated 3T3-L1 adipocytes were incubated with 100 µmol/L LA (L) or *t*10*c*12 CLA (C) **a** for 2 h, or **b** for 12 h, with or without sirtuin inhibitors sirtinol (SOL) or nicotinamide (NAM), or **c** with or without AMPK inhibitor compound C (Comp.C). **d** The effects of PPARγ agonist troglitazone (Tro) or PPARγ antagonist GW9662 (9662), in combination with LA or *t*10*c*12 CLA, on the acetylation of the p65 subunit of NF-κB was measured. Each bar in panels **a–d** represents the mean + SEM (n = 3) of three independent experiments. Means not sharing a common letter differ, *P* 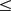
0.05.

The regulation of SIRT1 activity by AMPK and PPARγ was then assessed. A known deacetylation target of SIRT1 is Lys310 of the p65 subunit of NF-κB [38]. Therefore, we determined whether the p65 subunit of NF-κB changed its acetylation levels in response to *t*10*c*12 CLA. The acetylation level of the p65 subunit of NF-κB was not significantly changed by treatment with *t*10*c*12 CLA for 2 h (Fig. 6A). In contrast, the amount of acetylated p65 subunit of NF-κB was significantly less in adipocytes treated with *t*10*c*12 CLA for 12 h (Fig. 6B). When *t*10*c*12 CLA was used in combination with sirtinol or nicotinamide inhibitors of sirtuin activity, the amount of the acetylated p65 subunit of NF-κB was significantly higher relative to *t*10*c*12 CLA alone (Fig. 6 B). These drug inhibition results supported the premise that one or more members of the sirtuin family of protein deacetylases were involved in the deacetylation of the p65 subunit of NF-κB. Of the seven sirtuin family members, only SIRT1 and SIRT6 have a nuclear localization [51] that overlaps with the subcellular localization of acetylated NF-κB. Additionally, of the seven sirtuins, only SIRT1 is known to deacetylate the p65 subunit of NF-κB [38]. Therefore, these results established that one or more members of the sirtuins, presumably at least SIRT1, were responsible for the deacetylation of the p65 subunit of NF-κB, and verified that sirtuin deacetylase activity increased in *t*10*c*12 CLA treated adipocytes.

### AMPK and PPARγ affect SIRT1 activity

The involvement of AMPK in regulating SIRT1 activity was then measured using compound C, an inhibitor of AMPK. The amount of the acetylated p65 subunit of NF-κB significantly increased in a treatment using *t*10*c*12 CLA in combination with compound C, relative to the acetylation level produced by *t*10*c*12 CLA (Fig. 6C). This result indicated AMPK was directly or indirectly involved in regulating the SIRT1 deacetylation response to *t*10*c*12 CLA. The PPARγ agonist troglitazone was used to determine whether PPARγ affected the levels of acetylation of the p65 subunit of NF-κB. When used in combination with *t*10*c*12 CLA for 12 h, troglitazone-treated adipocytes significantly increased the amount of acetylated p65 subunit of NF-κB relative to the *t*10*c*12 CLA treatment, suggesting activated PPARγ interferes with SIRT1 deacetylation activity (Fig. 6D). Conversely, adipocytes treated with *t*10*c*12 CLA and the PPARγ antagonist GW9662 significantly reduced the amounts of acetylated p65 subunit of NF-κB relative to the *t*10*c*12 CLA treatment, suggesting inhibition of PPARγ increases SIRT1 deacetylation activity (Fig. 6D). Collectively, these results indicated that AMPK positively regulated, and PPARγ negatively regulated, SIRT1 activity *in vivo*, as measured by the deacetylation of the p65 subunit of NF-κB.

### SIRT1 increases its binding to PPARγ and NCoR1 in *t*10*c*12 CLA treated adipocytes

**Figure 7:**
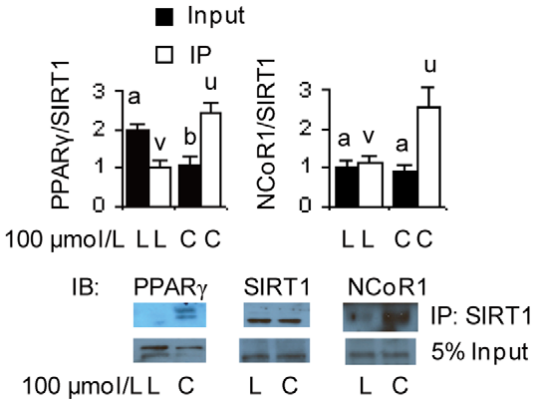
Treatment with *t*10*c*12 CLA increases the interaction of SIRT1 with PPARγ or NCoR1. Differentiated 3T3-L1 adipocytes were incubated with 100 µmol/L LA (L) or *t*10*c*12 CLA (C) for 12 h, and a portion of the nuclear extracts were immunoprecipitated with antibody to SIRT1 (IP: SIRT1). Representative immunoblots (IB) indicate the proteins detected from the nuclear extracts (5% input) or when the immunoprecipitated proteins were probed with antibodies to SIRT1, PPARγ, or NCoR1. Each bar represents the mean + SEM (n = 3) of three independent experiments. Means within each data type not sharing a common letter differ, *P* 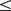
0.05.

A possible mechanism for cross regulation of SIRT1 and PPARγ is through participation within a common protein complex [35]. Using co-immunoprecipitation with a SIRT1 antibody, we observed that *t*10*c*12 CLA treatment resulted in a significant increase in the amount of a SIRT1/PPARγ protein complex relative to the amount of this complex in the LA control, despite an overall reduction in PPARγ protein levels in *t*10*c*12 CLA treated cells (Fig. 7). Again using the same co-immunoprecipitation method, we observed that the amount of a protein complex containing SIRT1 and NCoR1 also significantly increased in the presence of *t*10*c*12 CLA (Fig. 7). These results demonstrated that there was increased binding of SIRT1 to PPARγ and NCoR1 in *t*10*c*12 CLA treated adipocytes.

## Discussion

**Figure 8:**
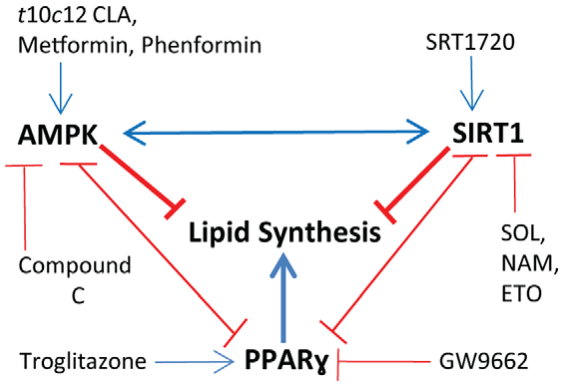
Summary diagram for proposed effects of AMPK, SIRT1, PPARγ, and their activators or inhibitors in 3T3-L1 adipocytes. AMPK and SIRT1 impair lipid synthesis and PPARγ activity (red lines with stop bars), and stimulate each other (blue arrows). PPARγ impairs AMPK and SIRT1 activity and stimulates lipid synthesis. The activating (blue arrows) or inhibiting (red line with stop bar) effects of the different chemical activators and inhibitors are also shown.

Here we demonstrated that SIRT1 activity was functionally involved in the TG loss response that occurred in *t*10*c*12 CLA-treated 3T3-L1 adipocytes. Our chemical inhibitor and activator studies indicated SIRT1 facilitated TG loss, as did AMPK, while PPARγ stimulated TG synthesis (summarized in Figure 8). Our assays of protein activities, modifications, and interactions supported this functional data and provided molecular evidence of cross regulation between SIRT1, AMPK and PPARγ in the response to *t*10*c*12 CLA. As discussed below, each of these proteins directly or indirectly affected the activity of the others.

In order to evaluate the effects of SIRT1 on specific pathways in lipid metabolism, we first established the effects of *t*10*c*12 CLA on lipid metabolism in our 3T3-L1 adipocyte system. The decrease in TG levels in *t*10*c*12 CLA-treated adipocytes was caused by a combination of reduced fatty acid biosynthesis, increased lipolysis, and increased fatty acid oxidation. Our finding that *t*10*c*12 CLA-treated adipocytes have increased p-AMPK levels and increased lipolysis and fatty acid oxidation is consistent with a report that activated AMPK increases lipolysis and fatty acid oxidation [22]. Our results were also consistent with the increased lipolysis and fatty acid oxidation, but not the increased fatty acid biosynthesis, reported in *t*10*c*12 CLA-treated 3T3-L1 adipocytes [6]. The latter difference may reflect differences in the specific cell cultures. The reduction in the rate of fatty acid synthesis was consistent with the reduced transcript levels of lipid biosynthetic enzymes such as ACC1, ACC2, and fatty acid synthase in mice and 3T3-L1 adipocytes [10], [15], possibly mediated by AMPK's ability to phosphorylate and inhibit SREBP1, a key regulatory factor of these genes [52]. Further, the reduction in the rate of fatty acid synthesis was consistent with increased phosphorylation of ACC, which inhibits ACC (both ACC-1 and ACC-2 isoforms) and thereby inhibits production of malonyl-CoA. Reduced concentrations of malonyl-CoA should reduce the rate of fatty acid biosynthesis and increase the rate of fatty acid oxidation, as attenuated levels of malonyl-CoA no longer inhibit carnitine palmitoyltransferase-mediated transport of fatty acids into mitochondria for β-oxidation [18], [19], [50]. Fatty acid synthesis was also likely reduced by AMPK's ability to phosphorylate and inhibit fatty acid synthase [18], [19]. Our studies indicated that SIRT1 primarily affected the rate of fatty acid biosynthesis as inhibition of SIRT1 did not significantly affect the rate of lipolysis or fatty acid oxidation in the response to *t*10*c*12 CLA. Whether SIRT1 regulates fatty acid biosynthesis primarily via differential modulation of the activities of AMPK and PPARγ or additionally through direct deacetylation of metabolic enzymes [53] requires additional research.

Our results strongly support an increase in SIRT1 deacetylase activity occurs *in vivo* in *t*10*c*12 CLA treated adipocytes. We used the acetylation levels of Lys310 of the p65 subunit of NF-κB as an indicator of SIRT1 deacetylase activity *in vivo*. NF-κB/p65 had reduced amounts of acetylation at Lys310 in *t*10*c*12 CLA-treated adipocytes, indicating increased deacetylase activity was occurring. However, in principle Lys310 of p65/NF-κB can be deacetylated by both class I/II or sirtuin deacetylases [38]. Results with the sirtuin-specific inhibitors indicated the majority of NF-κB/p65 deacetylation was accomplished by the sirtuin class of deacetylases. Of the seven sirtuin family members, only SIRT1 and SIRT6 have the nuclear localization [51] needed to deacetylate nuclear-localized NF-κB. Of these two candidates, SIRT1 is likely to be involved as SIRT1 physically binds to the p65 subunit of NF-κB and deacetylates it at Lys310 in human lung cells [38]. Further, our siRNA knockdown of SIRT1 indicates it is the major contributor to SIRT function in the *t*10*c*12 CLA response (Figure 1E). Therefore, our results strongly support an increase in the deacetylase activity of SIRT1 in *t*10*c*12 CLA-treated adipocytes, without ruling out minor contributions from other sirtuins.

The functional consequences of alterations in SIRT1, AMPK, and PPARγ activities suggested there was cross-regulation between these proteins (summarized in Figure 8). Therefore, evidence for changes in protein modifications and/or activity was investigated. In the case of SIRT1 affecting AMPK, an activator of SIRT1 increased AMPK activity, while inhibitors of SIRT1 reduced AMPK activity in *t*10*c*12 CLA-treated adipocytes. A possible pathway connecting SIRT1 to AMPK is a SIRT1/LKB1/AMPK axis by which SIRT1 can affect AMPK activity via deacetylation of protein kinase LKB1, which increases LKB1's ability to activate AMPK by phosphorylation [36], [37]. In the case of SIRT1 affecting PPARγ, we found treatment with *t*10*c*12 CLA caused more SIRT1 to bind to PPARγ and NCoR1. This is likely to inhibit PPARγ activity as the increased binding of SIRT1 to PPARγ and NCoR1 that occurred during fasting reduced PPARγ transcriptional activity [35]. Reduced PPARγ activity is consistent with the reduced transcription of lipogenic genes observed in *t*10*c*12 CLA treated adipocytes [10], [13], [15]. These results support a conclusion that SIRT1 stimulated AMPK activity and attenuated PPARγ activity in *t*10*c*12 CLA treated adipocytes.

In the case of AMPK affecting SIRT1, inhibition of AMPK with compound C reduced SIRT1 activity as measured by the deacetylation of p65/NF-κB. The mechanism is unclear but could include AMPK-mediated changes in fatty acid oxidation which affect the NAD^+^/NADH ratio that affects SIRT1 activity [43]. In the case of AMPK affecting PPARγ, AMPK was directly or indirectly responsible for the increased phosphorylated at Ser112 of PPARγ in *t*10*c*12 CLA treated adipocytes [29], as this effect was attenuated by the AMPK inhibitor compound C. Phosphorylation of PPARγ at Ser112 facilitates its SUMOylation at K107, and thereby decreasing its transactivation activity [26]. These results support a conclusion that AMPK stimulated SIRT1 activity and attenuated PPARγ activity in *t*10*c*12 CLA treated adipocytes.

In the case of PPARγ affecting AMPK and SIRT1, troglitazone, a PPARγ agonist, reduced the activities of AMPK and SIRT1. Conversely, GW9662, an antagonist of PPARγ, increased the activities of AMPK and SIRT1. These results demonstrate that PPARγ has a repressive effect on the activities of these proteins, which is consistent with the opposing roles of PPARγ's in stimulating lipid biosynthesis and the catabolic energy-generating roles of AMPK and SIRT1 [30], [54]. The mechanisms of how PPARγ affects AMPK and SIRT1 are unclear, despite the physical interaction between SIRT1 and PPARγ [35], [55]. Although the mechanisms are unclear, PPARγ affected the activity levels of SIRT1 and AMPK without changing the total amounts of these proteins in the response to *t*10*c*12 CLA. This suggests that PPARγ achieved these effects via a non-transcriptional mechanism. As such, our results support an emerging role for PPARγ in regulating non-genomic processes [27], [28].

We also used AMPK activators in addition to *t*10*c*12 CLA to manipulate AMPK activity and explore whether cross regulation of AMPK and PPARγ occurred in the absence of *t*10*c*12 CLA (summarized in Figure 8). Phenformin, a potent AMPK activator, caused a TG loss similar to that caused by *t*10*c*12 CLA treatment. Troglitazone, the most potent PPARγ agonist in our 3T3-L1 adipocyte system, attenuated the TG loss caused by phenformin. Conversely, GW9662, a PPARγ antagonist, increased the amount of TG loss when used with metformin, a moderate AMPK activator. This latter finding supports a hypothesis that both AMPK activation [9] and reduced PPARγ activity [3] are important for reducing TG levels. Taken together, these results support a hypothesis that cross regulation between AMPK and PPARγ also occurs in the absence of *t*10*c*12 CLA, and is therefore likely to generally occur between these proteins in adipocytes.

Both AMPK and SIRT1 play major roles in regulating cellular energy homeostasis and in response to caloric restriction [54], [56], [57]. The involvement of AMPK and SIRT1 in the response to *t*10*c*12 CLA is consistent with an overall similarity to cellular energy restriction. This is supported by the strong similarity of the whole genome transcriptional response of adipocytes treated with *t*10*c*12 CLA to the response caused by metformin [9], which affects the cellular AMP/ATP ratio [58], [59]. Similarly, phenformin, which also affects the cellular AMP/ATP ratio, caused TG losses similar to those caused by *t*10*c*12 CLA and caused a whole genome transcriptional response similar to that of *t*10*c*12 CLA-treated adipocytes [17]. Our results indicated SIRT1 activation and cross regulation of AMPK occurred at 12 h but not as early as 2 h, while AMPK was activated at 2 h or earlier [9]. This is consistent with the suggestion that AMPK activation is a critical early event [9]. The signaling pathways used by *t*10*c*12 CLA to activate AMPK remain unknown.

## Supporting Information

Figure S1 (TIF)

Figure S2 (TIF)

Figure S3 (TIF)
