# Supplementary figures and images for: Cross Regulation of Sirtuin 1, AMPK, and PPARγ in Conjugated Linoleic Acid Treated Adipocytes
Source: PLoS One. 2012 Nov 14;7(11):e48874. doi: 10.1371/journal.pone.0048874 (PMC3498327; doi:10.1371/journal.pone.0048874)

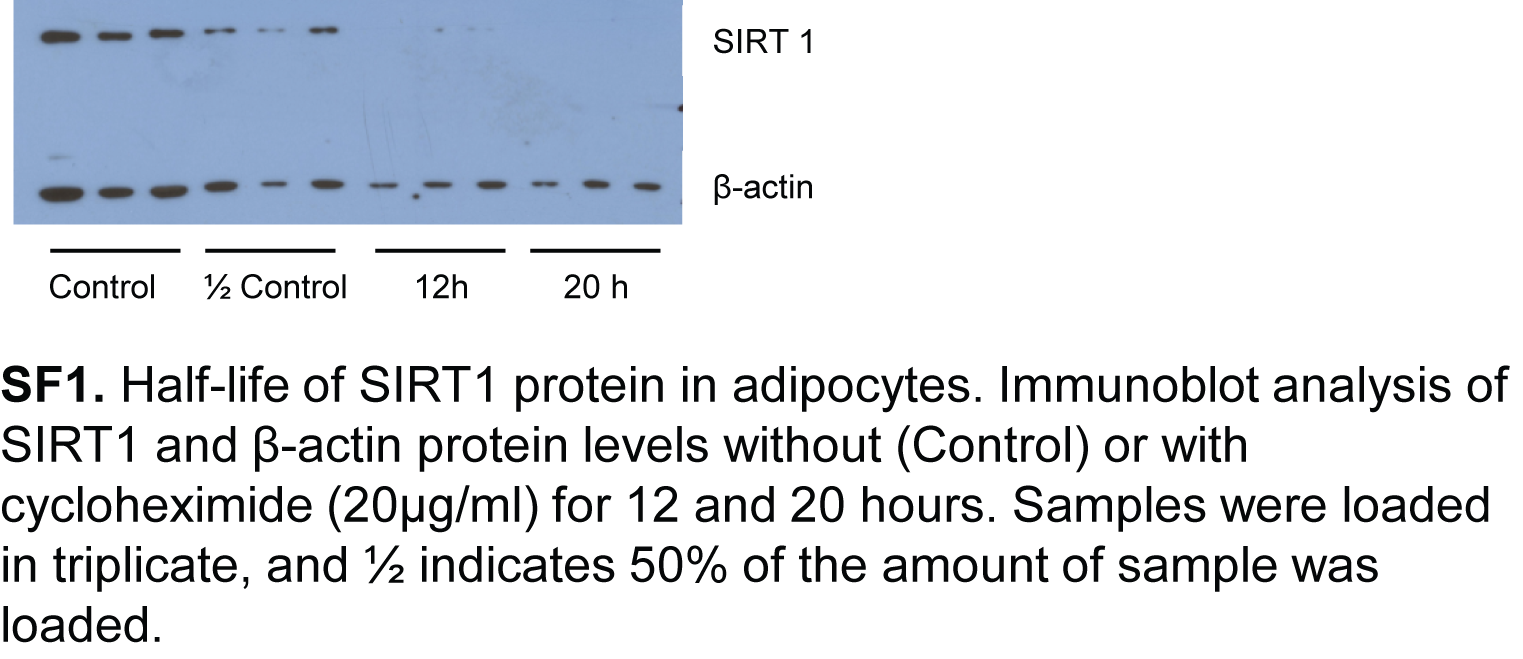

Supplement: Figure S1 — (TIF) [file pone.0048874.s001.tif]

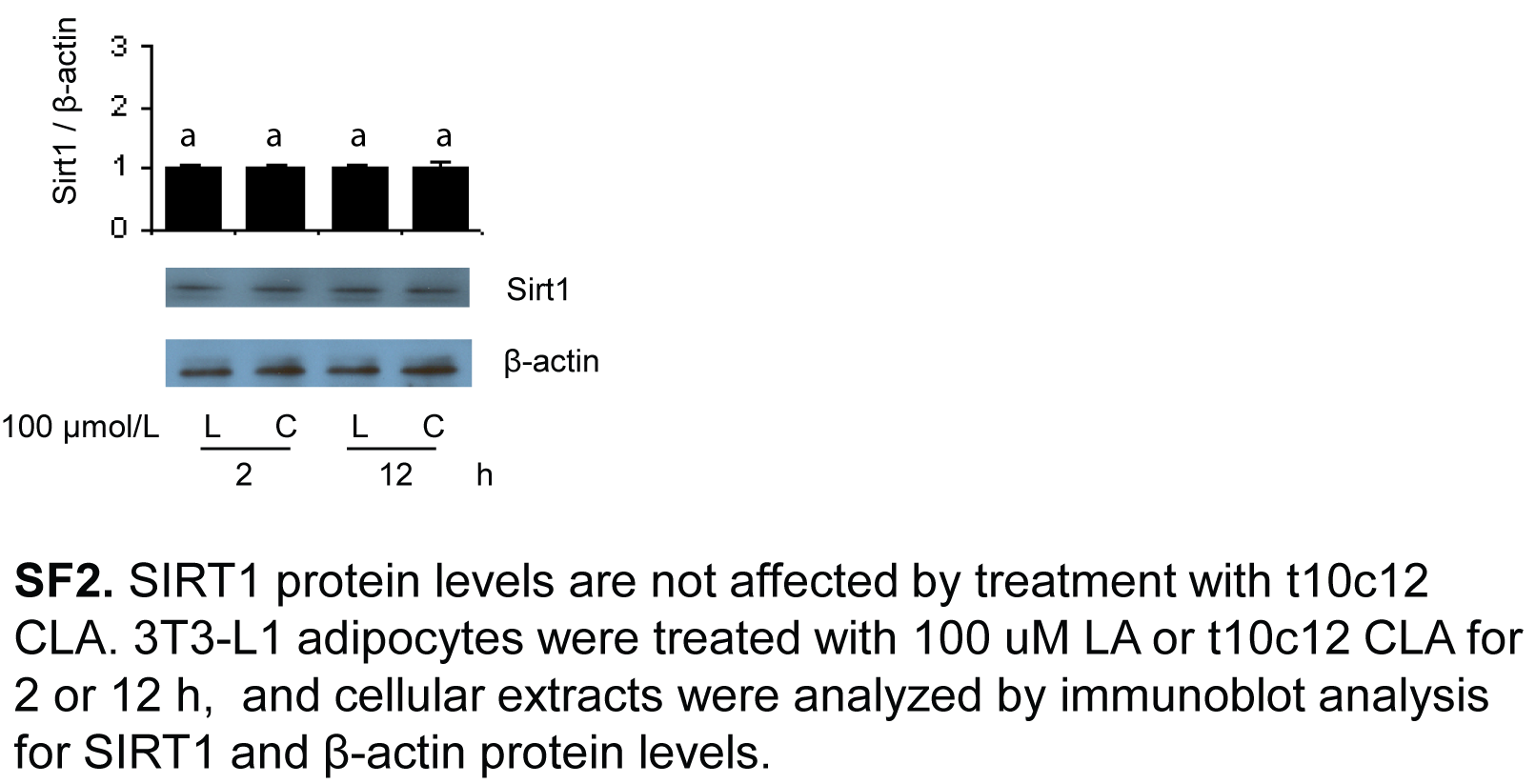

Supplement: Figure S2 — (TIF) [file pone.0048874.s002.tif]

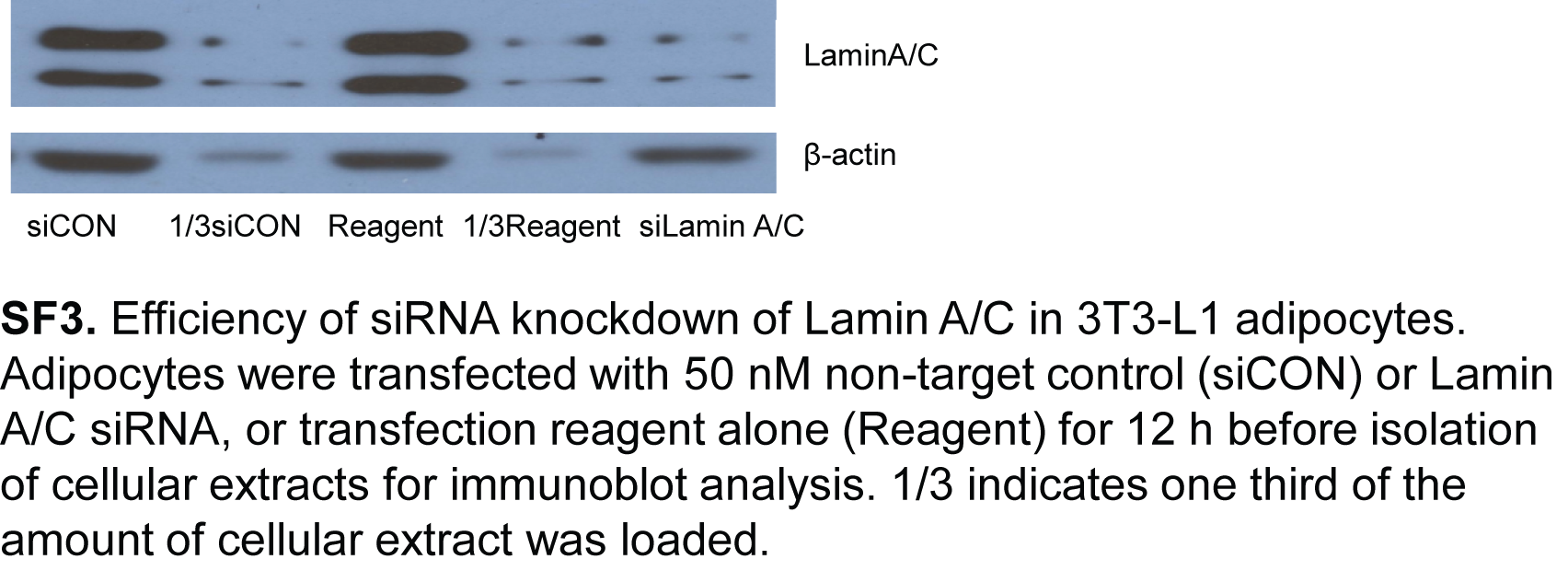

Supplement: Figure S3 — (TIF) [file pone.0048874.s003.tif]
